# Anxiety and depression symptoms in relation to body mass index, fruit and vegetable intake, and physical activity among nutrition students in Peru: a cross-sectional study

**DOI:** 10.3389/fnut.2026.1833831

**Published:** 2026-07-07

**Authors:** Jacksaint Saintila

**Affiliations:** Research Group on Nutritional Psychology and Public Health, Escuela de Posgrado, Universidad Peruana Unión, Lima, Peru

**Keywords:** anxiety, body mass index, depression, fruit and vegetable intake, lifestyle-related factors, mental health, physical activity, sleep duration

## Abstract

**Background:**

Anxiety and depression are common among university students and may negatively affect their wellbeing and academic performance. However, evidence regarding their co-occurrence and their relationship with BMI and lifestyle-related factors in Peruvian students remains limited. This study aimed to analyze the association between anxiety, depression, BMI, and lifestyle-related factors among nutrition students at a private university in northern Peru.

**Methods:**

A cross-sectional study was conducted with 405 university students. Anxiety and depressive symptoms were assessed using the Generalized Anxiety Disorder-2 (GAD-2) and the Patient Health Questionnaire-2 (PHQ-2). A cut-off score of ≥3 was used to identify clinically relevant symptoms. Data were collected on body mass index (BMI), sleep duration, physical activity (PA), and fruit and vegetable (F/V) consumption. Associations were evaluated using Poisson regression models with robust variance to estimate prevalence ratios (PR) and 95% confidence intervals (95% CI).

**Results:**

The prevalence of anxiety and depression symptoms was 57.3 and 26.4%, respectively. Anxiety symptoms was significantly associated with depression (aPR = 4.53; 95% CI: 2.63–7.80). Compared with normal weight, overweight and obesity were associated with a higher prevalence of anxiety (overweight: aPR = 1.22; 95% CI: 1.01–1.48; obesity: aPR = 1.43; 95% CI: 1.10–1.87) and depression (overweight: aPR = 1.27; 95% CI: 1.00–1.62; obesity: aPR = 1.61; 95% CI: 1.12–2.32). Engaging in PA ≥ 5 times per week was associated with a lower prevalence of depression (aPR = 0.35; 95% CI: 0.14–0.85), whereas consuming <5 servings/day of F/V was associated with a higher prevalence of depression (aPR = 1.52; 95% CI: 1.14–2.02).

**Conclusion:**

Anxiety and depression symptoms were highly prevalent and strongly interrelated. Excess body weight, low PA, and low F/V consumption were associated with poorer mental health indicators, highlighting the importance of integrated health-promotion strategies among university students.

## 1 Introduction

Symptoms of depression and anxiety represent a significant public health concern among university students, particularly those enrolled in health-related programs (1). Depression is characterized by persistent low mood, loss of interest or pleasure, and cognitive and somatic symptoms that impair daily functioning, whereas anxiety involves excessive worry, heightened arousal, and difficulties in emotional regulation (2). Studies conducted among Peruvian university students in the health sciences have reported that approximately 20–70% present depressive symptoms and 20–80% report anxiety symptoms, depending on the assessment tool, cut-off points, academic context, and timing during the pandemic (1, 3–5). Globally, health sciences students show average prevalence rates of around 35% for depression and 40–45% for anxiety (6, 7). Specifically, a study among nutrition students in Brazil reported a prevalence of 41% for anxiety and 20% for depression (8). Likewise, a multicenter study among medical students in northern Peru reported a prevalence of 61.8% for anxiety and 22.0% for depression (1). It is important to note that unhealthy lifestyle factors, such as poor diet quality, inadequate sleep duration and quality, sedentary behaviors, and elevated body mass index (BMI), have been associated with poorer mental health outcomes (9–13).

BMI is a widely used indicator to assess body weight in relation to height and to classify individuals as underweight, normal weight, overweight, or obese (14). Studies conducted in Peru (15) and in other regions worldwide have reported that between 25 and 35% of health sciences students are overweight or obese (16–18), despite the World Health Organization (WHO) recommendation of maintaining a BMI between 18.5 and 24.9 kg/m^2^ as a healthy range for adults (14). This situation may be partly explained by the adoption of unhealthy lifestyle behaviors, even among students with health-related training (15, 16). Regarding the relationship between excess body weight and mental health, evidence specific to nutrition students remains limited. One study conducted among dietetics students reported significant associations between BMI and mental health variables (10). Similarly, studies among medical students suggest a relationship between overweight or obesity and an increased risk of depressive and anxiety symptoms (11). However, most studies, both observational (19) and interventional (20), have focused on general university populations, and some have reported inconsistent findings or no direct association (21). Given the limited evidence in nutrition students, further research is needed to explore the relationship between BMI and mental health outcomes within this group.

Among health sciences students, physical activity (PA) is particularly relevant due to its documented association with both physical and psychological wellbeing, as well as its role in shaping future health professionals as promoters of healthy lifestyles. Nevertheless, a low prevalence of sufficient PA has been reported in this population; for instance, up to 84.7% of students have been found to spend more than 6 h per day in sedentary behaviors (22). Additionally, studies indicate that between 40 and 60% of university students worldwide do not meet the WHO recommendations for weekly moderate-to-vigorous physical activity, highlighting a substantial gap between guidelines and actual practice. The WHO recommends at least 150 min of moderate-intensity or 75 min of vigorous-intensity aerobic PA per week for adults to achieve substantial health benefits (23). There is consistent evidence showing that higher levels of PA are associated with lower levels of anxiety and depression among university students, including those in health-related fields (12, 13). Furthermore, recent studies conducted in Latin American university populations have reported that students engaging in regular moderate-to-vigorous PA exhibit significantly lower symptom scores of depression and anxiety compared to their less active peers (24–26) Given that these students may exhibit particular characteristics, such as greater concern about body weight, dietary norms, and perfectionism (27, 28), it is important to examine how PA relates to mental health in this specific group.

Sleep is a fundamental biological process that plays a critical role in emotional regulation and psychological wellbeing. Previous research has consistently identified sleep disturbances and short sleep duration as important correlates of anxiety and depressive symptoms among university students. Sleep deprivation has been associated with emotional dysregulation, increased stress reactivity, and alterations in neurotransmitter systems involved in mood regulation (29). Furthermore, anxiety and depressive symptoms may impair sleep quality and duration, suggesting a complex and potentially bidirectional relationship between sleep and mental health (30). Therefore, sleep-related factors should be considered when examining determinants of anxiety and depressive symptoms in young adults.

Fruit and vegetable (F/V) consumption is a fundamental component of a healthy diet and has been widely associated with both physical and mental health benefits (31–33). Growing evidence suggests that higher F/V intake is associated with lower levels of depression and anxiety, as well as greater psychological wellbeing (9, 34). These effects may be explained by the intake of micronutrients and bioactive compounds involved in mood regulation and in the reduction of inflammation and oxidative stress (31–33). However, as with other lifestyle factors, most studies have focused on general university populations, and evidence specifically targeting nutrition students remains scarce.

The relationships between lifestyle behaviors and mental health may be interpreted within broader motivational and psychological frameworks. Self-Determination Theory (SDT) posits that individuals’ wellbeing is influenced by the fulfillment of three basic psychological needs: autonomy, competence, and relatedness (35). In the university context, lifestyle behaviors such as physical activity, dietary patterns, and weight-related practices may reflect underlying motivational processes that shape students’ perceptions of self-regulation, health competence, and social belonging (36). Insufficient fulfillment of these needs may contribute to maladaptive coping strategies, including sedentary behavior, unhealthy eating patterns, or body dissatisfaction, which may in turn be associated with symptoms of anxiety and depression. Although the present study did not directly assess motivational constructs, SDT offers a useful integrative framework to contextualize the observed associations between BMI, physical activity, F/V intake, and mental health outcomes in this population.

Despite growing evidence on mental health among university students, limited research has simultaneously examined anxiety, depression, BMI, physical activity, and F/V intake within a theoretically informed framework in nutrition students, particularly in the Peruvian context.

Accordingly, informed by a broader motivational and behavioral perspective, this study aimed to examine the associations between symptoms of anxiety and depression and key anthropometric and lifestyle-related factors—including BMI, physical activity, and F/V intake—among nutrition students at a private university in northern Peru. Specifically, the study addressed the following research questions: (1) Are anxiety and depressive symptoms significantly associated in this population? (2) Is BMI associated with the prevalence of anxiety and depressive symptoms? (3) Are physical activity levels and F/V intake associated with anxiety and depressive symptoms? Based on prior empirical evidence and motivational theory, we hypothesized that (H1) anxiety and depressive symptoms would show a strong positive association; (H2) higher BMI would be associated with a greater prevalence of anxiety and depressive symptoms; (H3) lower levels of physical activity would be associated with higher prevalence of anxiety and depressive symptoms; and (H4) lower F/V intake would be associated with higher prevalence of depressive symptoms.

## 2 Materials and methods

### 2.1 Study design and sample size

A cross-sectional study was conducted among undergraduate students enrolled in the Nutrition program at a private university in Trujillo, Peru. Data collection took place at the beginning of the first academic semester of 2026 using an online questionnaire. Participants were selected through a non-probabilistic convenience sampling approach, whereby all students enrolled in the program were invited to voluntarily participate in the study through institutional communication channels. A total of 460 students were eligible and invited to participate. Of these, 405 students completed the questionnaire (response rate: 88.0%), and their data were included in the final analysis.

The sample size was estimated using *Free Statistics Calculators version 4.0* (37) a tool widely used for sample size calculation in regression models. For the regression analysis, a small effect size (0.10), a significance level of 0.05, and a statistical power of 0.80 were considered. In addition, relevant explanatory variables were included in the model, such as age, sex, BMI, sleep duration, PA, and F/V consumption. Based on these parameters, the minimum required sample size was 384 participants. However, a total of 405 university students participated in the study and completed the questionnaire, exceeding the estimated minimum sample size and ensuring adequate statistical power for the analyses performed. The inclusion criteria were being 18 years of age or older, being enrolled as a student in the Human Nutrition program, being registered in the corresponding academic semester, and providing voluntary consent to participate in the study. Questionnaires with incomplete responses or missing data in the main variables of interest were excluded.

### 2.2 Procedure

Data were collected using a structured questionnaire administered digitally through the Google Forms platform. The study protocol was previously reviewed and approved by the Research Ethics Committee of the Faculty of Health Sciences at Universidad Peruana Unión (Ref. 2026-CEUPeU-013). Prior to data collection, authorization was obtained from the academic authorities of the Nutrition program to conduct the survey among students. The questionnaire link was disseminated through institutional communication channels, including academic WhatsApp groups and institutional email lists. Additionally, during virtual class sessions, the researcher requested permission from course instructors to briefly present the study objectives and invite students to participate voluntarily in the survey. Before accessing the questionnaire, participants were provided with information regarding the study objectives, the voluntary nature of their participation, and the confidentiality of the data collected. Informed consent was obtained from all participants prior to completing the questionnaire.

### 2.3 Sociodemographic data

A data collection form was designed to gather sociodemographic information, including age (years), sex (female and male), region of origin (coast, highlands, jungle, and foreign), and marital status (single, married, cohabiting, divorced, and widowed).

### 2.4 Anxiety symptoms (GAD-2)

Anxiety symptoms were assessed using the Generalized Anxiety Disorder-2 (GAD-2), an ultra-brief scale developed by Kroenke et al. in 2007 (38) as a shortened version of the Generalized Anxiety Disorder-7 (GAD-7) to facilitate rapid screening of anxiety symptoms in clinical and epidemiological settings. The instrument consists of two items derived from the GAD-7: (1) “Feeling nervous, anxious, or on edge” and (2) “Not being able to stop or control worrying.” These items assess the frequency of symptoms over the past 2 weeks (38). Each item is scored on an ordinal scale from 0 (“not at all”) to 3 (“nearly every day”), yielding a total score ranging from 0 to 6. A cut-off point of ≥3 has demonstrated optimal performance for identifying probable cases of anxiety disorders. In the Peruvian context, the GAD-2 has shown adequate validity and reliability (Cronbach’s *α* = 0.80) in university populations (39). In the present study, the scale demonstrated good internal consistency, with a Cronbach’s α coefficient of 0.82.

### 2.5 Depressive symptoms (PHQ-2)

Depressive symptoms were assessed using the Patient Health Questionnaire-2 (PHQ-2), a brief instrument derived from the Patient Health Questionnaire-9 (PHQ-9), developed as a screening tool for the rapid detection of depressive symptoms in clinical and population-based settings. The instrument consists of two items that assess the core symptoms of depression: (1) “Feeling down, depressed, or hopeless” and (2) “Little interest or pleasure in doing things” (40). These items evaluate symptom frequency over the past 2 weeks, based on the question: “In the last 2 weeks, how often have you been bothered by any of the following problems?” (40). Each item is scored on an ordinal scale from 0 (“not at all”) to 3 (“nearly every day”), yielding a total score ranging from 0 to 6. According to previous validation studies, a cut-off point of ≥3 is the most commonly used threshold to identify clinically relevant depressive symptoms. In the Peruvian context, the PHQ-2 has demonstrated adequate psychometric properties, with acceptable internal consistency assessed using both Cronbach’s alpha (*α* = 0.75) and McDonald’s omega (*ω* = 0.76) in adult populations (41). In the present study, the PHQ-2 showed good internal consistency, with a Cronbach’s α coefficient of 0.79.

### 2.6 Sleep

Sleep duration was assessed using a single-item question: “On average, how many hours do you sleep per night?” Participants reported their average nightly sleep duration, and responses were subsequently categorized as <7 h/day, 7–9 h/day, and >9 h/day, according to international recommendations for young adults (42). The use of single-item measures to assess sleep duration has been widely employed in population-based studies and epidemiological surveys due to their simplicity and their adequate ability to estimate general sleep patterns in large samples (43).

### 2.7 Physical activity

Physical activity was assessed using a single-item question formulated as follows: “In a typical week, how often do you engage in physical activity?” Response options included never, 1–2 times per week, 3–4 times per week, and ≥5 times per week (44). The use of single-item measures to estimate general levels of PA has been widely applied in large-scale epidemiological studies, as it provides valid information on activity patterns while minimizing participant burden (45).

### 2.8 Fruit and vegetable consumption

F/V intake was assessed using a single-item question: “In a typical day, how many servings of fruits and vegetables do you consume?” Responses were subsequently categorized as <5 servings/day and ≥5 servings/day, in accordance with international recommendations for minimum F/V intake to promote health (46). This type of brief measure has been widely used in studies of health behaviors and epidemiological surveillance to estimate general dietary habits in adult and university populations (47).

### 2.9 BMI

Anthropometric data, including body weight and height, were self-reported by participants. BMI was calculated using the standard formula weight (kg)/height^2^ (m^2^). Subsequently, BMI was classified according to the WHO criteria into the following categories: underweight (<18.5 kg/m^2^), normal weight (18.5–24.9 kg/m^2^), overweight (25.0–29.9 kg/m^2^), and obesity (≥30 kg/m^2^) (48). Given that body weight and height were self-reported, measures were implemented to minimize potential information bias. Participants were informed about the importance of providing accurate data, and confidentiality was assured. In addition, standardized instructions were provided to guide participants in reporting their body weight and height as accurately as possible. They were asked to report their most recent known measurements or, if unavailable, their best estimate. The use of self-reported anthropometric measures has been widely applied in large-scale epidemiological studies and has demonstrated acceptable validity for estimating BMI (49).

### 2.10 Statistical analysis

Statistical analyses were performed using R software version 4.4.1. A descriptive analysis of the sample was conducted; continuous variables were summarized using means and standard deviations, while categorical variables were presented as frequencies and percentages. Anxiety and depression were analyzed both as continuous variables (total scores) and as categorical variables using clinically validated cut-off points (≥3 points). For bivariate analysis, continuous variables were compared using the Wilcoxon rank-sum test for independent samples, whereas categorical variables were analyzed using the chi-square test or Fisher–Freeman–Halton exact test when expected cell counts were small. A *p* < 0.05 was considered statistically significant.

Associations between anxiety and depression were estimated using Poisson regression models with robust variance, reporting prevalence ratios (PRs) and 95% confidence intervals (95% CIs). Both crude and adjusted models were fitted, including age, sex, region of origin, marital status, BMI, sleep duration, PA, and F/V consumption as covariates, selected *a priori* based on theoretical relevance. The relationship between anxiety and depression was examined bidirectionally, fitting models with depression as the dependent variable and anxiety as the independent variable, and vice versa. As a sensitivity analysis, continuous GAD-2 and PHQ-2 scores were used to assess the robustness of the findings. Multicollinearity was evaluated using the variance inflation factor (VIF), with values <5 indicating no problematic collinearity.

## 3 Results

The study comprised 405 nutrition students with a mean age of 25.2 years. Overall, most participants had a normal BMI, although a substantial proportion were classified as overweight or obese. Women represented slightly more than half of the sample, and the majority were single and from the coastal region. Regarding lifestyle characteristics, short sleep duration was common, and levels of physical activity were generally low, with a considerable proportion reporting no regular activity. Approximately half of the participants reported consuming fewer than five daily servings of fruits and vegetables. The detailed descriptive characteristics of the sample are presented in Table 1.

**Table 1:** Sociodemographic, anthropometric, lifestyle-related, and mental health characteristics of the study sample (*N* = 405).

Variable	M ± SD or n (%)
Age (years)	25.18 ± 7.23
BMI (kg/m^2^)	24.89 ± 6.32
BMI (categories)	
Underweight	5 (1.2)
Normal weight	253 (62.5)
Overweight	114 (28.1)
Obesity	33 (8.1)
GAD-2	3.00 ± 2.23
PHQ-2	1.97 ± 2.09
Sex	
Female	211 (52.1)
Male	194 (47.9)
Region of origin	
Coast	267 (65.9)
Highlands	63 (15.6)
Jungle	46 (11.4)
Foreign	29 (7.2)
Marital status	
Single	332 (82.0)
Married	45 (11.1)
Cohabiting	28 (6.9)
Sleep duration	
< 7 h/day	251 (62.0)
7–9 h/day	141 (34.8)
> 9 h/day	13 (3.2)
PA	
Never	110 (27.2)
1–2 times/week	168 (41.5)
3–4 times/week	79 (19.5)
≥ 5 times/week	48 (11.9)
F/V intake	
< 5 servings/day	203 (50.1)
≥ 5 servings/day	202 (49.9)

Data are presented as mean ± standard deviation (M ± SD) or frequency (percentage). GAD-2, Generalized Anxiety Disorder-2; PHQ-2, Patient Health Questionnaire-2; BMI, body mass index; PA, physical activity; F/V, fruit and vegetables.

The prevalence of clinically relevant anxiety symptoms (GAD-2 ≥ 3) was 57.3% (*n* = 232; 95% CI: 52.4–62.0), while the prevalence of clinically relevant depressive symptoms (PHQ-2 ≥ 3) was 26.4% (*n* = 107; 95% CI: 22.4–30.9).

As part of a sensitivity analysis, the association between anxiety and depression was evaluated using continuous GAD-2 and PHQ-2 scores. In adjusted models, each one-point increase in anxiety score was associated with a significant increase in the prevalence of depression, whereas each one-point increase in depression score was associated with a higher prevalence of anxiety. These findings were consistent in both direction and magnitude with those obtained using categorical clinical definitions.

Table 2 summarizes the distribution of sociodemographic, anthropometric, lifestyle-related, and mental health variables according to anxiety status. Students with anxiety exhibited markedly higher depressive symptom scores compared to those without anxiety (*p* < 0.001). Consistently, clinically relevant depressive symptoms were strongly associated with anxiety, with a substantial proportion of students with depressive symptoms also presenting clinically relevant anxiety symptoms. Significant differences were observed according to physical activity levels, with anxiety being more frequent among students reporting no regular physical activity. In contrast, no statistically significant differences were identified for age, sex, region of origin, marital status, BMI, sleep duration, or F/V consumption.

**Table 2:** Distribution of sociodemographic, anthropometric, lifestyle-related, and mental health variables according to the presence of anxiety (*N* = 405).

Variable	Category	Without anxiety (GAD-2 < 3) n (%)	With anxiety (GAD-2 ≥ 3) n (%)	*p*-value
Age (M ± SD)	–	25.18 ± 7.19	25.17 ± 7.28	0.880
PHQ-2 (M ± SD)	–	0.77 ± 1.23	2.86 ± 2.16	<0.001
PHQ-2 categorized	<3 points	160 (53.7)	138 (46.3)	<0.001†
	≥3 points	13 (12.1)	94 (87.9)	
Sex	Female	93 (44.1)	118 (55.9)	0.634
	Male	80 (41.2)	114 (58.8)	
Region of origin	Coast	119 (44.6)	148 (55.4)	0.608
	Highlands	27 (42.9)	36 (57.1)	
	Jungle	16 (34.8)	30 (65.2)	
	Foreign	11 (37.9)	18 (62.1)	
Marital status	Single	136 (41.0)	196 (59.0)	0.403
	Married	22 (48.9)	23 (51.1)	
	Cohabiting	15 (53.6)	13 (46.4)	
BMI	Underweight	4 (80.0)	1 (20.0)	0.109†
	Normal weight	101 (39.9)	152 (60.1)	
	Overweight	56 (49.1)	58 (50.9)	
	Obesity	12 (36.4)	21 (63.6)	
Sleep duration	<7 h/day	101 (40.2)	150 (59.8)	0.208
	7–9 h/day	68 (48.2)	73 (51.8)	
	>9 h/day	4 (30.8)	9 (69.2)	
PA	Never	31 (28.2)	79 (71.8)	0.001
	1–2 times/week	78 (46.4)	90 (53.6)	
	3–4 times/week	36 (45.6)	43 (54.4)	
	≥5 times/week	28 (58.3)	20 (41.7)	
F/V intake	<5 servings/day	83 (40.9)	120 (59.1)	0.519
	≥5 servings/day	90 (44.6)	112 (55.4)	

Values are presented as mean ± standard deviation or n (%), as appropriate. Percentages were calculated by row. Continuous variables were compared using the Mann–Whitney U test. Categorical variables were compared using the chi-square test or Fisher–Freeman–Halton exact test when expected cell counts were small. GAD-2 ≥ 3 indicates clinically relevant anxiety symptoms. PHQ-2 ≥ 3 indicates clinically relevant depressive symptoms. BMI, body mass index; PA, physical activity; F/V, fruits and vegetables. †Fisher–Freeman–Halton exact test.

Table 3 displays the distribution of sociodemographic, anthropometric, lifestyle-related, and mental health variables according to depressive status. Students with depression showed substantially higher anxiety scores than those without depression (*p* < 0.001). Significant differences were also observed across BMI categories, with a higher proportion of depressive symptoms among students with excess body weight. Physical activity was likewise associated with depression, as lower activity levels were linked to a greater prevalence of depressive symptoms. In contrast, no statistically significant differences were found according to age, sex, region of origin, marital status, sleep duration, or F/V consumption.

**Table 3:** Distribution of sociodemographic, anthropometric, lifestyle-related, and mental health variables according to the presence of depression (*N* = 405).

Variable	Category	Without depression (PHQ-2 < 3) n (%)	With depression (PHQ-2 ≥ 3) n (%)	*p*-value
Age (M ± SD)	–	25.38 ± 7.81	24.61 ± 5.32	0.947
GAD-2 (M ± SD)	–	2.38 ± 2.08	4.75 ± 1.63	<0.001
Sex	Female	160 (75.8)	51 (24.2)	0.338
	Male	138 (71.1)	56 (28.9)	
Region of origin	Coast	200 (74.9)	67 (25.1)	0.711
	Highlands	45 (71.4)	18 (28.6)	
	Jungle	31 (67.4)	15 (32.6)	
	Foreign	22 (75.9)	7 (24.1)	
Marital status	Single	238 (71.7)	94 (28.3)	0.527
	Married	36 (80.0)	9 (20.0)	
	Cohabiting	24 (85.7)	4 (14.3)	
BMI	Underweight	3 (60.0)	2 (40.0)	0.046†
	Normal weight	197 (77.9)	56 (22.1)	
	Overweight	78 (68.4)	36 (31.6)	
	Obesity	20 (60.6)	13 (39.4)	
Sleep duration	<7 h/day	182 (72.5)	69 (27.5)	0.144
	7–9 h/day	109 (77.3)	32 (22.7)	
	>9 h/day	7 (53.8)	6 (46.2)	
PA	Never	61 (55.5)	49 (44.5)	<0.001
	1–2 times/week	125 (74.4)	43 (25.6)	
	3–4 times/week	69 (87.3)	10 (12.7)	
	≥5 times/week	43 (89.6)	5 (10.4)	
F/V intake	<5 servings/day	147 (72.4)	56 (27.6)	0.674
	≥5 servings/day	151 (74.8)	51 (25.2)	

Values are presented as mean ± standard deviation or n (%), as appropriate. Percentages were calculated by row. Continuous variables were compared using the Mann–Whitney U test. Categorical variables were compared using the chi-square test or Fisher–Freeman–Halton exact test when expected cell counts were small. PHQ-2 ≥ 3 indicates clinically relevant depressive symptoms. GAD-2 ≥ 3 indicates clinically relevant anxiety symptoms. BMI, body mass index; PA, physical activity; F/V, fruits and vegetables. †Fisher–Freeman–Halton exact test.

In the multivariable Poisson regression model with robust variance, BMI and depressive symptoms were independently associated with anxiety. Although BMI categories were not significantly associated with anxiety in the bivariate analysis, students classified as overweight and obese showed a higher prevalence of anxiety after adjustment compared with those with normal BMI. Depressive symptoms showed a strong and consistent association with anxiety. In separate adjusted models, both the continuous PHQ-2 score and the categorical definition of clinically relevant depressive symptoms were significantly associated with a greater prevalence of anxiety in the adjusted model. No statistically significant associations were observed for age, sex, region of origin, marital status, sleep duration, physical activity, or F/V consumption (Table 4).

**Table 4:** Factors associated with anxiety symptoms (GAD-2 ≥ 3) based on Poisson regression with robust variance.

Variable	Category	Crude PR	95% CI	*p*-value	Adjusted PR	95% CI	*p*-value
Age (years)	–	1.00	0.99–1.01	0.993	1.01	0.99–1.02	0.437
Sex	Female	1.00	–	–	1.00	–	–
	Male	1.05	0.89–1.24	0.564	1.01	0.86–1.18	0.924
Region of origin	Coast	1.00	–	–	1.00	–	–
	Foreign	1.12	0.83–1.52	0.466	1.16	0.87–1.55	0.297
	Jungle	1.18	0.93–1.49	0.179	1.07	0.85–1.36	0.570
	Highlands	1.03	0.81–1.31	0.803	1.00	0.81–1.25	0.971
Marital status	Married	1.00	–	–	1.00	–	–
	Single	1.16	0.86–1.56	0.345	1.26	0.93–1.71	0.134
	Cohabiting	0.91	0.56–1.48	0.701	1.21	0.78–1.89	0.393
BMI	Normal weight	1.00	–	–	1.00	–	–
	Underweight	0.33	0.06–1.93	0.220	0.91	0.35–2.39	0.851
	Overweight	0.85	0.69–1.04	0.115	1.22	1.01–1.48	0.041
	Obesity	1.06	0.80–1.40	0.684	1.43	1.10–1.87	0.009
Sleep duration	<7 h/day	1.00	–	–	1.00	–	–
	7–9 h/day	0.87	0.72–1.05	0.137	0.91	0.77–1.08	0.283
	>9 h/day	1.16	0.80–1.69	0.444	0.87	0.48–1.59	0.648
PA	Never	1.00	–	–	1.00	–	–
	1–2 times/week	0.75	0.62–0.90	0.002	0.91	0.76–1.10	0.335
	3–4 times/week	0.76	0.60–0.96	0.020	1.10	0.84–1.43	0.505
	≥5 times/week	0.58	0.41–0.83	0.003	0.84	0.58–1.21	0.348
F/V intake	≥5 servings/day	1.00	–	–	1.00	–	–
	<5 servings/day	1.07	0.90–1.26	0.456	0.98	0.82–1.18	0.851
PHQ-2	Continuous score	1.20	1.16–1.23	<0.001	1.19	1.15–1.23	<0.001
PHQ-2	<3 points	1.00	–	–	1.00	–	–
	≥3 points	1.90	1.65–2.18	<0.001	1.78	1.52–2.09	<0.001

PR, prevalence ratio; 95% CI = 95% confidence interval. The PHQ-2 was included as a continuous variable; additionally, the scale was analyzed as a categorical variable using the standard cut-off of ≥3 points. The continuous and categorical versions of the same scale were not included simultaneously in the same model but were evaluated in separate models to avoid redundancy and collinearity. The adjusted model included age, sex, region of origin, marital status, BMI, sleep duration, PA, F/V intake, and PHQ-2 score. BMI, body mass index; PA, physical activity; F/V, fruits and vegetables.

In the adjusted Poisson regression model with robust variance (Table 5), BMI, physical activity, F/V intake, and anxiety were independently associated with depressive symptoms. Students classified as overweight or obese exhibited a higher prevalence of depression compared with those with normal BMI. Physical activity showed an inverse association with depressive symptoms, with lower prevalence observed among students engaging in regular activity across increasing frequency categories. Although F/V intake was not significantly associated with depressive symptoms in the bivariate analysis, consuming fewer than five daily servings was associated with a higher prevalence of depression after adjustment. In separate adjusted models, anxiety symptoms were the factor most strongly associated with depressive symptoms, both when assessed as a continuous GAD-2 score and when classified according to the ≥3 cut-off for clinically relevant anxiety symptoms.

**Table 5:** Factors associated with depressive symptoms (PHQ-2 ≥ 3) based on Poisson regression with robust variance (*N* = 405).

Variable	Category	Crude PR	95% CI	*p*-value	Adjusted PR	95% CI	*p*-value
Age (years)	–	1.00	0.99–1.01	0.947	1.00	0.98–1.02	0.876
Sex	Female	1.00	–	–	1.00	–	–
	Male	1.19	0.86–1.65	0.285	1.08	0.80–1.46	0.612
Region of origin	Coast	1.00	–	–	1.00	–	–
	Foreign	0.96	0.48–1.90	0.904	0.98	0.50–1.93	0.955
	Jungle	1.30	0.80–2.11	0.287	1.15	0.70–1.89	0.577
	Highlands	1.14	0.72–1.81	0.569	1.08	0.67–1.75	0.743
Marital status	Married	1.00	–	–	1.00	–	–
	Cohabiting	0.71	0.24–2.10	0.541	0.91	0.30–2.78	0.872
	Single	1.42	0.77–2.60	0.263	1.36	0.70–2.66	0.362
BMI	Normal weight	1.00	–	–	1.00	–	–
	Underweight	1.81	0.60–5.42	0.291	0.81	0.21–3.15	0.763
	Overweight	1.43	1.00–2.04	0.050	1.27	1.00–1.62	0.049
	Obesity	1.78	1.10–2.88	0.019	1.61	1.12–2.32	0.010
Sleep duration	<7 h/day	1.00	–	–	1.00	–	–
	7–9 h/day	0.83	0.57–1.19	0.303	0.89	0.65–1.22	0.463
	>9 h/day	1.40	0.68–2.86	0.358	0.95	0.26–3.47	0.938
PA	Never	1.00	–	–	1.00	–	–
	1–2 times/week	0.57	0.40–0.82	0.003	0.72	0.53–0.98	0.035
	3–4 times/week	0.28	0.15–0.51	<0.001	0.31	0.16–0.59	<0.001
	≥5 times/week	0.23	0.09–0.57	0.002	0.35	0.14–0.85	0.021
F/V intake	≥5 servings/day	1.00	–	–	1.00	–	–
	<5 servings/day	1.09	0.79–1.51	0.594	1.52	1.14–2.02	0.004
Anxiety (GAD-2)	Continuous score	1.55	1.40–1.71	<0.001	1.53	1.36–1.72	<0.001
Anxiety (GAD-2)	<3 points	1.00	–	–	1.00	–	–
	≥3 points	5.39	3.12–9.30	<0.001	4.53	2.63–7.80	<0.001

PR, prevalence ratio; 95% CI = 95% confidence interval. The GAD-2 was included as a continuous variable; additionally, the scale was analyzed as a categorical variable using the standard cut-off of ≥3 points. The continuous and categorical versions of the same scale were not included simultaneously in the same model but were evaluated in separate models to avoid redundancy and collinearity. The adjusted model included age, sex, region of origin, marital status, BMI, sleep duration, PA, F/V intake, and GAD-2 score. BMI, body mass index; PA, physical activity; F/V, fruits and vegetables.

## 4 Discussion

The present study examined the association between symptoms of anxiety, depression, BMI, and lifestyle-related factors among nutrition students from a private university in northern Peru. Overall, the findings revealed a high prevalence of anxiety and depressive symptoms in this population, as well as a strong relationship between both conditions. In addition, relevant associations were observed between mental health and anthropometric and lifestyle-related factors, particularly BMI, PA, and F/V intake. These findings suggest the presence of an interconnected pattern between mental health, nutritional status, and health-related behaviors among university students.

In the present study, a high prevalence of anxiety and depressive symptoms was observed among nutrition students. These findings are consistent with previous research conducted in health sciences students in Peru. For instance, a study conducted in Brazil among nutrition students reported that 41% presented anxiety symptoms and 20% reported depressive symptoms (8), prevalence rates comparable to those observed in our sample. Similarly, other studies in Peruvian university students have documented high levels of anxiety and depression, highlighting that students in health-related fields may be particularly vulnerable due to the academic and emotional demands of their training (50). In our sample, these findings may be explained by the fact that nutrition students are often exposed to social and professional norms related to body weight, healthy eating, and body image (27, 28), which may increase levels of anxiety and depression (51) due to heightened self-demand and concerns about health and physical appearance. Additionally, factors inherent to university life, such as changes in sleep habits, irregular dietary patterns, insufficient PA, and adaptation to new academic responsibilities, may contribute to the development or exacerbation of anxiety and depressive symptoms in this population (52).

A second key finding of this study was the strong association between anxiety and depression, as students with anxiety symptoms showed a substantially higher prevalence of depressive symptoms. This pattern of comorbidity is consistent with findings reported in Peruvian medical students (1), in whom a significant association between both conditions has also been documented. Similarly, studies conducted among health sciences students have described anxiety and depression as frequently co-occurring conditions in this population (7). For example, Hoying et al. (53), observed that students enrolled in health sciences programs exhibit a notable coexistence of anxiety and depressive symptoms from the early stages of their professional training, suggesting that these mental health problems may emerge early in academically demanding environments. One possible explanation is that both conditions share common emotional and cognitive mechanisms, such as persistent worry, rumination, intolerance of uncertainty, and psychological overload in response to academic demands, factors frequently described among health sciences students (7). In the case of nutrition students, this relationship may be further reinforced by the academic pressure inherent to a health-oriented discipline.

Another relevant finding of this study was the association between BMI and mental health. Although BMI was not significantly associated with anxiety in the bivariate analysis, students with overweight and obesity showed a higher prevalence of anxiety after multivariable adjustment. In addition, students with overweight or obesity showed a higher prevalence of depressive symptoms compared with those with normal weight. These results are consistent with findings from a cross-sectional study in dietetics students, which reported a notable coexistence of anxiety, depression, and stress symptoms in this population, as well as associations between eating attitudes and BMI (10). Similarly, a study conducted among medical students found that overweight and obesity were significantly associated with symptoms of depression, anxiety, and stress, even after accounting for mediating factors such as academic burnout and internet addiction (11).

However, given the cross-sectional design of the present study, the direction of this relationship cannot be established. Although excess body weight has been linked to inflammatory and metabolic processes that may influence mood regulation (54), it is also possible that anxiety and depressive symptoms contribute to weight changes through mechanisms such as emotional eating, appetite dysregulation, reduced motivation for physical activity, or alterations in sleep patterns (55). Therefore, the observed association may reflect a bidirectional or reciprocal relationship rather than a unidirectional effect. These findings may be explained by the fact that obesity is associated with a state of chronic low-grade inflammation, characterized by increased levels of pro-inflammatory cytokines such as interleukin-6 (IL-6) and tumor necrosis factor-alpha (TNF-*α*), which can influence brain circuits involved in mood regulation (56). In addition, visceral adipose tissue may act as an endocrine organ that alters the secretion of adipokines, such as leptin and adiponectin, which are involved in appetite regulation and stress response.

Furthermore, the study revealed an inverse association between the frequency of physical activity and depressive symptoms. In the adjusted models, higher PA frequency was associated with a lower prevalence of depressive symptoms, whereas the association between PA and anxiety did not remain statistically significant after adjustment. Indeed, a large study conducted among Serbian medical students showed that those with more severe symptoms of depression and anxiety were significantly less likely to engage in PA, highlighting physical inactivity as a predictor of mental health problems (12). Similarly, another study reported that PA directly reduced symptoms of depression and anxiety among university students (13). Nevertheless, these findings should be interpreted with caution. While regular physical activity has been associated with better mental health outcomes, depressive symptoms may also reduce motivation, energy levels, and engagement in physical activity, suggesting a potentially bidirectional relationship (57). Therefore, it cannot be determined whether lower PA precedes depressive symptoms or whether depressive symptoms lead to lower PA levels in this population. Promoting regular PA is essential as a key intervention strategy in the university setting, not only to improve physical health but also as a fundamental component in the prevention and management of mental health problems in this population.

These effects may be explained by several physiological mechanisms. Physical exercise increases levels of brain-derived neurotrophic factor, which promotes neuroplasticity and resilience against depressive and anxiety disorders (58). In addition, it modulates neurotransmitters such as serotonin, dopamine, and endorphins, which are involved in mood regulation and the generation of wellbeing (58). Moreover, PA contributes to reducing systemic inflammation, improving the regulation of the hypothalamic–pituitary–adrenal (HPA) axis, and decreasing oxidative stress in the brain, processes that have been linked to better mental health outcomes (59). These biological effects, together with behavioral benefits such as improved sleep and enhanced self-esteem, may help explain the inverse association observed between PA and depressive symptoms among university students (59).

Sleep duration was not significantly associated with anxiety or depressive symptoms in the adjusted models. This non-significant finding may be explained by the fact that sleep was assessed mainly in terms of duration, while other sleep-related dimensions, such as sleep quality, insomnia symptoms, bedtime irregularity, and perceived restfulness, may be more closely linked to psychological distress among university students (29, 30). Therefore, future studies should include validated measures of sleep quality and circadian patterns to better understand the role of sleep in the mental health of nutrition students.

Finally, the present study found that students who consumed fewer than five servings of F/V per day had a higher prevalence of depressive symptoms after multivariable adjustment. Although this association was not observed in the bivariate analysis, the adjusted finding is consistent with previous studies reporting an association between higher F/V intake and better mental health in both young and adult populations (9, 34). For instance, a population-based study conducted in Peru found that individuals in the lowest tertile of F/V consumption had nearly twice the prevalence of depressive symptoms compared to those in the highest tertile (9). Although this study was not limited to health sciences students, it reflects a broader pattern relevant to the Peruvian population (9). In addition, multinational surveys including university students have also reported an inverse relationship between fruit consumption and depression, suggesting that these findings may extend to health sciences students (34). Although direct evidence in nutrition students remains limited, the protective effect of F/V intake against depression observed in similar populations supports its potential relevance in this group. This may be explained by the fact that F/V are rich sources of vitamins, minerals, fiber, and bioactive compounds such as polyphenols and carotenoids (31), which may influence biological processes involved in mood regulation. These include the reduction of oxidative stress, modulation of inflammatory pathways (32, 33), and participation in neurotransmitter synthesis, particularly through nutrients such as folate and vitamin C (60).

Cultural context may also influence the interpretation and generalizability of these findings. Peru, like many Latin American countries, has been described as relatively collectivistic, with strong emphasis on family interdependence and social cohesion (61). In collectivistic contexts, mental health experiences and health-related behaviors may be shaped by social expectations, family norms, and relational obligations (62). For instance, body weight concerns and dietary behaviors may be influenced not only by individual preferences but also by shared family eating practices and sociocultural standards regarding appearance and health (63). Furthermore, cultural dimensions such as verticality and horizontality may modulate how psychological distress is experienced and expressed (64). In more vertically oriented societies, hierarchical academic environments and performance expectations may intensify stress and self-demand among health sciences students. Therefore, the strength of the associations observed in this study may differ in more individualistic or horizontally structured educational systems, where autonomy and self-expression are more strongly emphasized.

### 4.1 Limitations and future directions

This study has several limitations that should be considered when interpreting the findings. First, the cross-sectional design does not allow for the establishment of causal relationships between anxiety, depression, BMI, and the lifestyle-related factors assessed. Second, the data were collected through self-report, which may introduce recall or social desirability bias, particularly for variables such as body weight, height, PA, and F/V intake. However, previous studies have reported adequate agreement between self-reported and measured BMI (49, 65), as well as the validity of brief measures to assess lifestyle behaviors such as PA, sleep, and dietary intake in epidemiological studies (42–45, 47, 66), allowing for reasonably accurate estimates in large populations. Therefore, although these measures may involve some degree of imprecision, their use is widely accepted and does not invalidate the overall interpretation of the findings.

In addition, the use of brief screening instruments such as the GAD-2 and PHQ-2 represents a limitation of the present study, as these tools are designed to detect symptoms rather than provide clinical diagnoses of mental disorders. Indeed, the assessment of clinical conditions ideally requires evaluation by a mental health professional, which is considered the gold standard (67). Nevertheless, both instruments have been validated and widely used in epidemiological and screening studies (38, 39, 41, 68), enabling the identification of clinically relevant symptoms in large populations, although without replacing a formal diagnosis.

Furthermore, the sample consisted of students from a single private university, which may limit the generalizability of the findings to other university populations or sociocultural contexts. Finally, other potentially relevant variables, such as academic stress, overall diet quality, social support, or pre-existing health conditions, were not assessed and may have acted as confounding factors. Future studies should employ longitudinal or prospective designs to better assess the directionality of the observed associations, as well as incorporate more detailed and objective measures of lifestyle behaviors and nutritional status. In addition, including larger and more diverse samples and exploring potential mediating and moderating mechanisms would contribute to a better understanding of the interplay between psychological, nutritional, and behavioral factors in university students.

### 4.2 Implications of the study

Despite the aforementioned limitations, the findings of this study have important implications for public health and the university community as a whole, particularly in health-related disciplines. These results highlight the need to implement early detection strategies and comprehensive approaches to address mental health among university students. In this context, health promotion programs should adopt integrated frameworks that simultaneously consider both physical and mental health.

Moreover, healthy lifestyle behaviors may serve as key tools in the prevention and management of mental health problems. Universities could play a pivotal role by implementing institutional interventions that promote regular PA, nutrition education, and access to healthy food environments as part of student wellbeing strategies. Finally, given that the sample consisted of nutrition students, the findings underscore the importance of strengthening academic training not only in technical knowledge but also in the adoption of healthy behaviors and self-care practices. This is essential to ensure that future professionals are able to act as credible health promoters, maintaining consistency between theoretical knowledge and personal health practices.

## 5 Conclusion

In conclusion, symptoms of anxiety and depression were highly prevalent and strongly associated with each other among nutrition students in our sample. In addition, excess body weight was associated with a higher prevalence of anxiety and depressive symptoms in the adjusted models, whereas regular PA showed an inverse association with depressive symptoms, and low F/V intake was associated with a higher prevalence of depression. These findings highlight the coexistence of psychological, anthropometric, and lifestyle-related factors and underscore the importance of implementing comprehensive strategies aimed at promoting healthy behaviors and mental wellbeing within the university setting.

## Data Availability

The raw data supporting the conclusions of this article will be made available by the authors, without undue reservation.
